# Activation of Peroxisome Proliferator-Activated Receptor-β/δ (PPARβ/δ) in Keratinocytes by Endogenous Fatty Acids

**DOI:** 10.3390/biom14060606

**Published:** 2024-05-21

**Authors:** Bokai Zhu, Xiaoyang Zhu, Michael G. Borland, Douglas H. Ralph, Christopher R. Chiaro, Kristopher W. Krausz, James M. Ntambi, Adam B. Glick, Andrew D. Patterson, Gary H. Perdew, Frank J. Gonzalez, Jeffrey M. Peters

**Affiliations:** 1 Department of Veterinary and Biomedical Sciences, The Center for Molecular Toxicology and Carcinogenesis, The Pennsylvania State University, University Park, PA 16802, USA; bzhu@pitt.edu (B.Z.); xzz145@psu.edu (X.Z.); mborland@commonwealth.edu (M.G.B.); dougralph1@gmail.com (D.H.R.); cxc223@psu.edu (C.R.C.); abg11@psu.edu (A.B.G.); adp117@psu.edu (A.D.P.); ghp2@psu.edu (G.H.P.); 2 Department of Biochemistry, Microbiology and Molecular Biology, The Pennsylvania State University, University Park, PA 16802, USA; 3 Department of Genetics, The Pennsylvania State University, University Park, PA 16802, USA; 4 Laboratory of Metabolism, Center for Cancer Research, National Cancer Institute, Bethesda, MD 20892, USA; krauszk@intra.nci.nih.gov (K.W.K.); gonzalef@mail.nih.gov (F.J.G.); 5 Department of Nutritional Sciences, University of Wisconsin-Madison, Madison, WI 53706, USA; jmntambi@wisc.edu; 6 Department of Biochemistry, University of Wisconsin-Madison, Madison, WI 53706, USA

**Keywords:** peroxisome proliferator-activated receptor, ligands, cell cycle, fatty acids, UVB-induced non-melanoma skin cancer

## Abstract

Nuclear hormone receptors exist in dynamic equilibrium between transcriptionally active and inactive complexes dependent on interactions with ligands, proteins, and chromatin. The present studies examined the hypothesis that endogenous ligands activate peroxisome proliferator-activated receptor-β/δ (PPARβ/δ) in keratinocytes. The phorbol ester treatment or HRAS infection of primary keratinocytes increased fatty acids that were associated with enhanced PPARβ/δ activity. Fatty acids caused PPARβ/δ-dependent increases in chromatin occupancy and the expression of angiopoietin-like protein 4 (*Angptl4*) mRNA. Analyses demonstrated that stearoyl Co-A desaturase 1 (*Scd1*) mediates an increase in intracellular monounsaturated fatty acids in keratinocytes that act as PPARβ/δ ligands. The activation of PPARβ/δ with palmitoleic or oleic acid causes arrest at the G2/M phase of the cell cycle of HRAS-expressing keratinocytes that is not found in similarly treated HRAS-expressing *Pparb/d*-null keratinocytes. HRAS-expressing *Scd1*-null mouse keratinocytes exhibit enhanced cell proliferation, an effect that is mitigated by treatment with palmitoleic or oleic acid. Consistent with these findings, the ligand activation of PPARβ/δ with GW0742 or oleic acid prevented UVB-induced non-melanoma skin carcinogenesis, an effect that required PPARβ/δ. The results from these studies demonstrate that PPARβ/δ has endogenous roles in keratinocytes and can be activated by lipids found in diet and cellular components.

## 1. Introduction

Peroxisome proliferator-activated receptors (PPARs) are ligand-activated transcription factors. Three subtypes of PPARs exist, PPARα, PPARβ/δ, and PPARγ, each with some unique yet overlapping functions [1]. After ligand binding, PPARs heterodimerize with retinoid X receptor (RXR) causing the recruitment of other proteins used to remodel chromatin and alter the expression of PPAR target genes (e.g., histone acetyl transferases, RNA polymerase) [2]. Cells respond to specific ligands/signals and modulate cellular activities to maintain homeostasis by interacting with PPARs. Transcriptional regulation mediated by PPARs is dynamic. There are several dynamic mechanisms that modulate nuclear receptor activity including the following: (1) those that impact the availability of endogenous/exogenous ligands, (2) those that influence the relative expression of the receptor, and (3) those that modulate accessory proteins for chromatin remodeling [3,4,5,6]. For example, non-esterified fatty acids released from adipose and transported to the liver during periods of fasting are endogenous ligands for PPARα [7]. In this mechanism, the ligand activation of PPARα by endogenous fatty acids increases the expression of target genes encoding lipid transporters, lipid binding proteins, and fatty acid-catabolizing enzymes that facilitate the production of energy from lipid substrates to meet the needs of the cells during periods of fasting [7]. This PPARα-specific mechanism is particularly critical to meet the energy demands of cells during periods of fasting [8], a cellular state that predominates daily in most species.

The relative expression of PPARs has been used to help determine important roles of these transcription factors in different cells and tissues. For example, PPARα expression is relatively high in hepatocytes, and this is reflected by the requirement of this receptor for the oxidation of fatty acids to generate energy in the liver, a tissue that is central in the homeostatic regulation of lipid metabolism [7]. In contrast, PPARβ/δ is constitutively expressed at a relatively high level in epithelial cells including those in the intestine and skin, in particular in keratinocytes [9,10]. Based on studies using synthetic ligands and transgenic models, it is now recognized that PPARβ/δ has important functional roles in epithelial cells [11,12]. The ligand activation of PPARβ/δ inhibits the proliferation of both mouse primary keratinocytes and human keratinocytes by inducing terminal differentiation through a PPARβ/δ-dependent mechanism [12]. The topical application of a PPARβ/δ ligand is also chemopreventive against chemically induced non-melanoma skin cancer through a PPARβ/δ-dependent mechanism that targets premalignant tumors as a decrease in keratoacanthomas is only noted in ligand-treated wild-type mice but not in *Pparb/d*-null mice [13,14,15]. Interestingly, the inhibition of cell proliferation by PPARβ/δ in keratinocytes is greater in cells expressing high levels of HRAS [13] mediated by PPARβ/δ-dependent negative selection against HRAS-expressing cells by inducing a mitotic block at the G2/M phase of the cell cycle [16].

Synthetic ligands and genetic manipulations in cells, tissues, and animal models have also been instrumental for elucidating the role(s) of PPARs. However, the identification and functional characterization of endogenous PPAR ligands remain elusive [17]. There is evidence indicating that keratinocytes exhibit constitutive transcriptional activity of PPARβ/δ in the absence of any exogenous ligands [6]. The expression of more than four hundred genes is altered when PPARβ/δ is ablated in mouse primary keratinocytes [6]. Moreover, previous studies suggested that an endogenous PPARβ/δ ligand is produced in keratinocytes after treatment with phorbol ester [18]. For these reasons, the hypothesis that an endogenous PPARβ/δ ligand exists and can be identified and functionally characterized in keratinocytes was examined in the present study.

## 2. Materials and Methods

### 2.1. Keratinocyte Culture

Two-day-old neonates from either *Pparb/d* wild-type, *Pparb/d*-null [19], *Scd1* wild-type, or *Scd1*-null mice [20] were used to isolate primary keratinocytes and cultured as previously described [21]. As previous studies suggested that an endogenous PPARβ/δ ligand is produced in keratinocytes after treatment with phorbol ester (phorbol-12-myristate-13-acetate; TPA) [18], 80–90% confluent keratinocytes were cultured in medium containing 25 ng TPA/mL for eight hours as described previously [18]. Four 100 mm culture dishes were used per treatment. After this treatment, cells were trypsinized, and the cytosol was isolated after differential centrifugation (100,000× *g*) for one hour.

### 2.2. HPLC Fractionation

The cytosolic fraction was diluted with 1 volume cytosol/2 volumes solvent (hexane/ethyl acetate; 1:1), vortexed, and then centrifuged (3000 rpm). Two hundred milligrams of anhydrous sodium sulfite was added to each sample, then dried under nitrogen, and resuspended in 99% hexane, 1% isopropanol, 0.1% acetic acid. These lipid-enriched fractions were then used to separate compounds using high-performance liquid chromatography (HPLC). HPLC purifications were performed using a Waters system, consisting of a Waters 600E multi-solvent delivery unit and controller coupled with a Waters 996 photodiode array detector (Waters, Milford, MA, USA). The system was integrated and operated using Waters Millenium^32^ software (version 3.20). Normal-phase HPLC purification was performed on a LiChrosphere 5 µm Silica-60 (4.6 × 250 mm) column (Supelco, Bellefonte, PA, USA) using a hexane/isopropanol/acetic acid solvent system [989:10:1 (*v*/*v*/*v*)] applied at 1 mL/min with a linear gradient increase in isopropanol concentration of 2.5%/min over 30 min. Fractions were collected every minute, dried under nitrogen, and resuspended in 100% ethanol before the examination of PPARβ/δ activity.

### 2.3. Quantitative Polymerase Chain Reaction (qPCR)

Primary mouse keratinocytes were isolated from 2-day-old neonates as described previously [21]. Keratinocytes were cultured in low-calcium (0.05 mM) Eagle’s minimal essential medium with 8% chelexed fetal bovine serum at 37 °C and 7% CO_2_ [21]. The keratinocytes were cultured until they were approximately 80% confluent and were then treated with either 0.2 μM GW0742 (positive control), 100 μM linoleic acid, 100 μM oleic acid, or 100 µM palmitoleic acid. After 8 h of treatment, total mRNA was isolated from the cells using TRIZOL and following the manufacturer’s recommended protocol (Invitrogen, Carlsbad, CA, USA). The mRNA encoding *Angiopoietin-like 4* (*Angptl4*) was quantified using quantitative real-time polymerase chain reaction (qPCR) analysis. The cDNA was generated using 2.5 µg total RNA with the MultiScribe Reverse Transcriptase kit (Applied Biosystems, Foster City, CA, USA). Primers were designed for real-time PCR using the Primer Express software (Version 3.0, Applied Biosystems, Foster City, CA, USA). The sequence and GenBank accession number for the forward and reverse primers used to quantify mRNAs was *Angptl4* (NM_020581) forward, 5′-TTCTCGCCTACCAGAGAAGTTGGG-3′, and reverse, 5′-CATCCACAGCACCTACAACAGCAC-3′. The mRNA was normalized to the gene encoding glyceraldehyde 3-phosphate dehydrogenase (*Gapdh*; BC083149) using the following primers: forward, 5′-GGTGGAGCCAAAAGGGTCAT-3′, and reverse, 5′-GGTTCACACCCATCACAAACAT-3′. qPCR reactions were carried out using SYBR green PCR master mix (Finnzymes, Espoo, Finland) in the iCycler and detected using the MyiQ Real-Time PCR Detection System (Bio-Rad Laboratories, Hercules, CA, USA). The following conditions were used for PCR: 95 °C for 15 s, 94 °C for 10 s, 60 °C for 30 s, and 72 °C for 30 s and repeated for 45 cycles. The PCR included a no-template control reaction to control for contamination and/or genomic amplification. All reactions had >95% efficiency. The relative expression levels of mRNA were normalized to *Gapdh* and analyzed for statistical significance using a one-way analysis of variance (Prism 5.0, GraphPad Software Inc., La Jolla, CA, USA).

### 2.4. ChIP Assays

*Pparb/d* wild-type or *Pparb/d*-null primary keratinocytes were treated for 3 h with vehicle (DMSO), GW0742 (0.2 μM), palmitoleic acid (100 μM), or oleic acid (100 µM). Cross-linking was performed using 1% formaldehyde for 10 min with gentle agitation, followed by the addition of glycine to a final concentration of 125 mM for an additional 10 min of gentle agitation. Cells were washed twice with PBS before the addition of lysis buffer (50 mM Tris-HCl pH 8, 1% SDS, 10 mM EDTA, and protease inhibitor cocktail). The DNA was sheared to obtain 500–1500 base pair fragments with the Diagenode Bioruptor™ (Diagenode, Sparta, NJ, USA). Protein A agarose (Santa Cruz Biotechnology, Dallas, TX, USA) beads were blocked with a solution of 1 μg/μL bovine serum albumin and 0.1 μg/μL salmon sperm DNA (Invitrogen) for 1 h prior to use. The sheared chromatin (2 units of 260 nm quantified DNA per immunoprecipitation) was precleared for 1 h with blocked protein A agarose before being immunoprecipitated with specific antibodies for rabbit IgG (Santa Cruz Biotechnology), acetylated histone H4 (Upstate Biotechnology, Charlottesville, VA, USA), or PPARβ/δ [9]. After 3 h, immune complexes were captured by the addition of blocked protein A agarose and were incubated overnight. The recovered beads were washed three times with a low-salt wash buffer (20 mM Tris-HCl pH 8, 2 mM EDTA, 0.1% sodium deoxycholate, 1% Triton-X, 150 mM NaCl, and protease inhibitor cocktail), once with a high-salt wash buffer (20 mM Tris-HCl pH 8, 2 mM EDTA, 0.1% sodium deoxycholate, 1% Triton-X, 500 mM NaCl, and protease inhibitor cocktail), and once with TE8 (10 mM Tris-HCl pH 8, 1 mM EDTA). The immune complexes were released by the addition of elution buffer (100 mM NaHCO_3_, 1% SDS), and the cross-links were reversed by overnight incubation at 65 °C. Immunoprecipitated DNA was purified by phenol/chloroform/isoamyl alcohol (25:24:1) extraction and subjected to real-time PCR analysis for occupancy at a response element of the known PPARβ/δ target gene angiopoietin-like protein 4 (*Angptl4*). The primer set for *Angptl4* was designed based on a previous identification of PPREs in the intron 3 coding sequence [22,23]. The primers for *Angptl4* were 5′-CTAGCCAAGTAGAGGAAAGTTCAGAGC-3′ (forward) and 5′-CCAATCCCTCGGGCAGCTAGC-3′ (reverse). Real-time PCR reactions were carried out as previously described in the RNA analysis section. Each PCR reaction included a no-template control to detect contamination, and all PCR reactions had greater than 85% efficiency. The specific values were normalized to treatment inputs and verified to be greater than rabbit IgG controls. Accumulation was determined based on fold accumulation as normalized to the control treatment.

### 2.5. HRAS Infection

The *Hras* retrovirus was generated from ψ2 producer cells as described previously [24]. The virus titer was determined to be between 1 and 2 × 10^7^ transforming units/mL using an NIH-3T3 focus-forming assay. Primary keratinocytes from newborn mice were prepared and cultured as previously described [21]. Keratinocytes were infected with the *Hras* retrovirus for two days at an estimated multiplicity of infection (M.O.I) of 1–12. Cells were subsequently cultured in control medium or medium containing palmitoleic acid, oleic acid, or the positive control GW0742.

### 2.6. Reporter Assays

Previously described plasmids containing the ligand-binding domain of mouse or human PPARβ/δ fused to the DNA binding domain of the yeast transcription factor Gal4 under the control of the SV40 promoter and the UAS-firefly luciferase reporter under the control of the Gal4 DNA response element were used to examine ligand activity [25]. Luciferase activity was measured using a luciferase reporter assay kit (Promega, Madison, WI, USA) and a Turner TD-20/20 Luminometer (Turner BioSystems, Sunnyvale, CA, USA) following the manufacturer’s recommended procedures. Luciferase activity was normalized to the beta-galactosidase activity of each sample. The fold induction of normalized luciferase activity was calculated relative to vehicle-control cells (DMSO) and represents the mean of three independent samples per treatment group.

### 2.7. Quantitative Western Blot Analyses

Cell lysates and samples used for Western blots, and immunoprecipitations were prepared as previously described [9,26]. Western blot analysis using radioactive detection methods was performed as previously described [9]. The primary antibodies used were as follows: Acyl CoA carboxylase (ACC); fatty acid synthase (FAS); fatty acid desaturase1/2 (FADS1, FADS2); very-long-chain fatty acid elongase 6 (ELOVL6); cytochrome-b5 reductase1 (CYB5R1); and stearoyl CoA desaturase1 (SCD1), (Santa Cruz Biotechnology, Santa Cruz, CA, USA), or anti-β-ACTIN (Rockland, Gilbertsville, PA, USA). The anti-PPARβ/δ antibody was previously described [9]. Original figures can be found in Supplementary Materials. 

### 2.8. Flow Cytometry

Control and HRAS-expressing *Scd1* wild-type or *Scd1*-null keratinocytes were cultured with or without GW0742, oleic acid, or palmitoleic acid for twenty-four hours and were stained with bromodeoxyuridine (BrdU) and/or propidium iodide and analyzed for cell cycle progression as previously described [16,27,28]. The percentage of cells at each phase of the cell cycle was determined with FCS Express software (version 4.00). 

### 2.9. Coulter Counting and BrdU Labeling of Keratinocytes

Control and HRAS-expressing *Scd1* wild-type and *Scd1*-null keratinocytes were cultured with or without GW0742, oleic acid, or palmitoleic acid for six days as described above. The average cell number was determined using a Coulter counter as previously described [29]. After five days, cells were labeled with BrdU for twenty-four hours and immunohistochemical analysis was performed to determine the average number of BrdU-labeled cells as previously described [29].

### 2.10. GC/MS

*Pparb/d* wild-type keratinocytes, with or without HRAS infection, were cultured for seventy-two hours. Cells were trypsinized, homogenized, and fatty acids were quantified using GC/MS as previously described [30].

### 2.11. Topical Tetradecanoylphorbol-13-Acetate (TPA)Treatment in Wild-Type and Scd1-Null Mice

*Scd1* wild-type and *Scd1*-null female mice aged 6–8 weeks, on an Sv/129 genetic background, were used for these studies [20]. Mice were shaved to remove back hair, and twenty-four hours later, either 25 µg of TPA (Sigma Chemical Co., St. Louis, MO, USA) dissolved in 200 µL of acetone or 200 µL of acetone was applied to the shaved area. Forty-eight hours post-TPA application, mice were euthanized, and the skin was removed and snap-frozen in liquid nitrogen. A section of skin was removed and placed in 10% phosphate buffered formalin for immunohistochemistry to assess the relative immunoreactivity for PCNA, keratin 5, or the nucleus as previously described. Skin samples were fixed with phosphate buffered formalin, embedded in paraffin, and sectioned at 5 μm thickness. Hematoxylin and eosin staining was performed using standard methods. Immunofluorescence staining was performed as previously described [29]. The following primary antibodies were used: anti-keratin 5 or anti-PCNA. Slides were kept in dark containers. Samples were washed three times and incubated with secondary antibodies at room temperature. The following secondary antibodies were used: anti-rabbit, anti-mouse, and anti-rat conjugated to AlexaFluor488 or AlexaFluor647. Stained slides were mounted in the mounting reagent containing DAPI for nuclei staining. The quantification of positive/negative cells was conducted manually using ImageJ (version 1.46).

### 2.12. UV-Induced Skin Cancer Using Pparb/d-Null Crossed with SKH Mice

Female SKH1 mice (*Pparβ/δ*^+/+^ or *Pparβ/δ*^−/−^) were UVB-irradiated (180 mJ/cm^2^) 3 times per week for 25 weeks. The amount and frequency of UVB irradiation as well as the timeframe for this study was designed based on methods used in a previously published UVB-induced tumor study [28]. UV-emitting lights that produce wavelengths in the UVB and UVC range were used for this study. UVC light was specifically prevented from reaching the mouse by using a filter that reflects wavelengths higher than UVB. The time of irradiation exposure was calculated prior to each UVB- irradiation by using the intensity of the emitted UVB light which was measured with a UVB-specific radiometer. Irradiation was immediately followed by a topical dorsal application of 200 µL of vehicle control (acetone), the highly specific PPARβ/δ ligand (1.0 µM GW0742), or 25 µM oleic acid. The rationale for the concentration of GW0742 and oleic acid used was based on previous studies showing that these concentrations are in the range that can activate PPARβ/δ [31,32]. The topical dorsal application of control or ligand solution started in week 1. The lesion incidence, multiplicity, and volume were measured and recorded once per week. The lesion incidence for each mouse is defined as the week when the first measurable lesion appeared. The lesion multiplicity for each mouse is defined as the total number of lesions found weekly. Within 6 h after the last irradiation of week 25, mice were euthanized by overexposure to carbon dioxide and cervical dislocation. The sample sizes ranged from 7 to 11 mice per treatment group. Data were analyzed for statistical significance using ANOVA and the Bonferroni post hoc test (Prism 10.0, GraphPad Software, San Diego, CA, USA). Differences between treatment groups were considered significant when *p* ≤ 0.05.

## 3. Results

### 3.1. PPARβ/δ Activity Is Increased in the Cytosol of Keratinocytes Post-TPA Treatment

**Figure 1:**
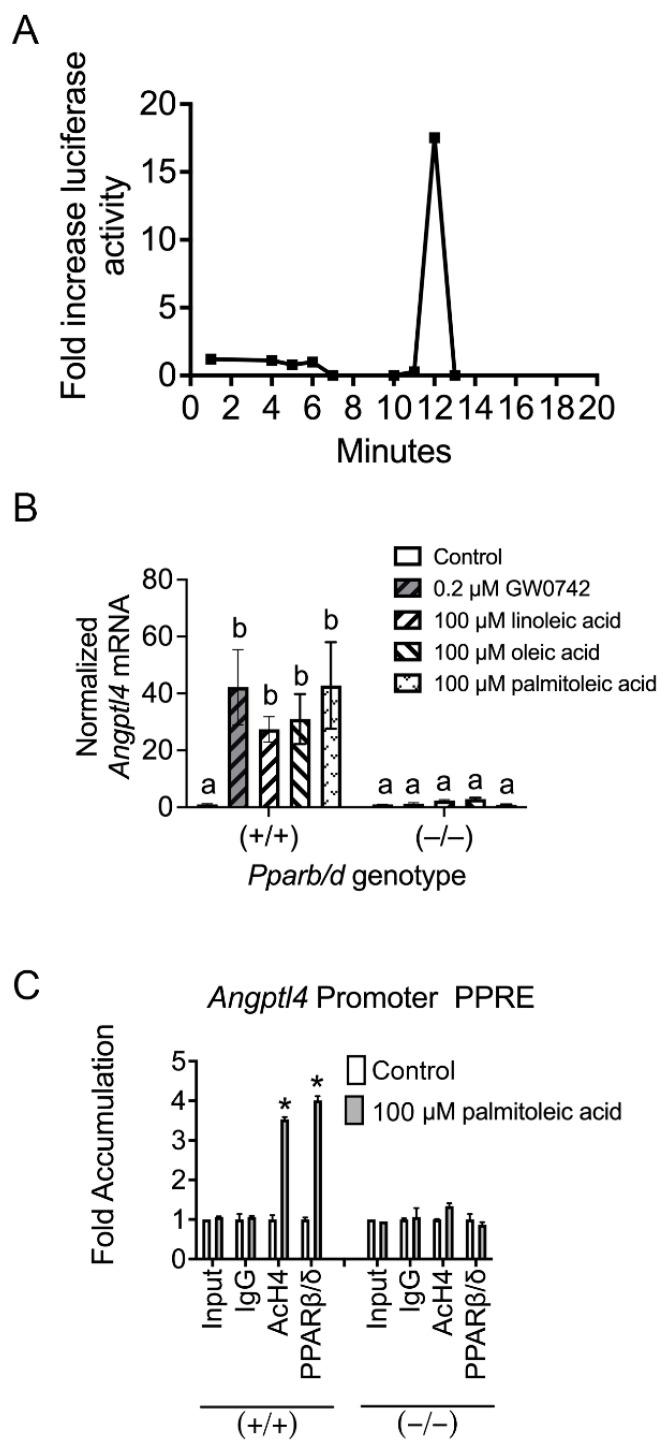
Phorbol ester (TPA) increases PPARβ/δ transcriptional activity in keratinocytes. (**A**) A reporter assay was used to detect PPARβ/δ activity in cytosolic isolates obtained from mouse primary keratinocytes after fractionation using HPLC. (**B**) Fatty acids known to be released by TPA treatment in wild-type keratinocytes increase the PPARβ/δ-dependent expression of *Angptl4* mRNA, and this effect is absent in similarly treated *Pparb/d*-null keratinocytes. *n* = 5 independent biological replicates. (**C**) A ChIP assay showing increased promoter occupancy on known *Angptl4* PPRE by palmitoleic acid in wild-type keratinocytes (+/+), an effect lacking in similarly treated *Pparb/d*-null keratinocytes (–/–), *n* = 5 independent biological replicates. Values represent the mean ± SD. Values with different letters are significantly different, *p* ≤ 0.05. * Significantly different than control, *p* ≤ 0.05.

Previous studies suggest that endogenous PPARβ/δ ligand(s) are produced in keratinocytes after treatment with TPA [18]. Since TPA activates phospholipases causing the release of fatty acids in keratinocytes [33], the organic fraction of cytosol obtained from either control mouse primary keratinocytes or mouse keratinocytes treated acutely with TPA were examined. Consistent with this past study [18], one fraction of organic keratinocyte cytosol caused an increase in PPARβ/δ reporter activity compared to controls (Figure 1A). Since TPA activates phospholipases causing the release of fatty acids in keratinocytes including linoleic and oleic acids [33], keratinocytes were cultured in linoleic, oleic, or palmitoleic acid, and the expression of the PPARβ/δ target gene, angiopoietin-like protein-4 (*Angplt4*), was measured. The expression of *Angptl4* mRNA was increased in wild-type primary keratinocytes in response to the synthetic ligand GW0742 and with linoleic acid, oleic acid, and palmitoleic acid compared to controls (Figure 1B). This effect was mitigated in *Pparb/d*-null keratinocytes (Figure 1B). Further, ChIP analyses demonstrated that palmitoleic acid increased the occupancy of PPARβ/δ on the *Angplt4* PPRE, and this effect was not observed in similarly treated *Pparb/d*-null keratinocytes (Figure 1C).

### 3.2. HRAS Activates PPARβ/δ

**Figure 2:**
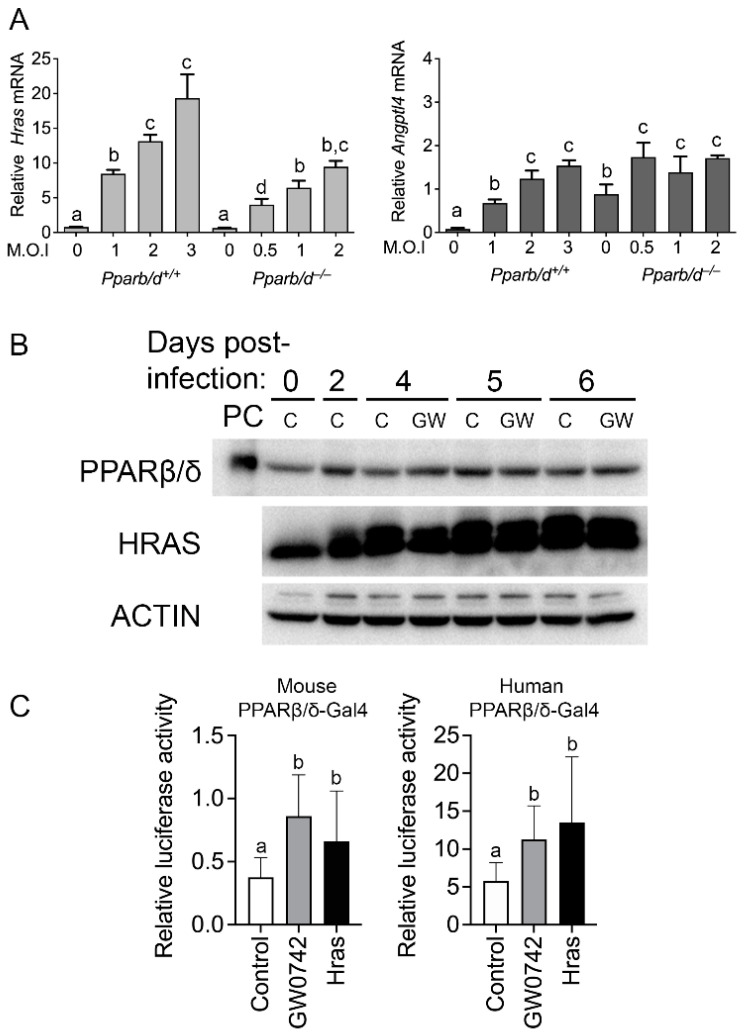
Activated HRAS increases PPARβ/δ transcriptional activity. (**A**) qPCR of *Hras* (**left panel**) or *Angptl4* (**right panel**) in mock-infected or HRAS-infected *Pparb/d* wild-type and *Pparb/d*-null keratinocytes with increasing M.O.I. *n* = 6 independent biological replicates. (**B**) Representative Western blot analysis of PPARβ/δ expression in HRAS-infected wild-type keratinocytes without GW0742 (**C**) or treated with GW0742 (GW). PC: positive control (COS cell lysate). (**C**) Luciferase assay in primary keratinocytes transiently transfected with either mouse (left panel) or human (right panel) PPARβ/δ-GAL4 fusion protein and UAS-luciferase plasmids. *n* = 6 independent biological replicates. Values represent mean ± SD. Values with different letters are significantly different, *p* ≤ 0.05.

TPA activates PKCα and other proteins including HRAS [34,35]. Thus, the impact of HRAS in keratinocyte signaling was examined. Interestingly, wild-type keratinocytes expressing HRAS exhibit an increased expression of *Angptl4* mRNA compared to controls, and this effect was muted in *Pparb/d*-null keratinocytes (Figure 2A,B). Interestingly, the basal expression of *Angptl4* mRNA was higher in *Pparb/d*-null keratinocytes compared to wild-type keratinocytes (Figure 2A), consistent with past studies showing that the basal expression of this gene is constitutively negatively regulated by PPARβ/δ [36]. The expression of PPARβ/δ was not influenced by HRAS or GW0742 (Figure 2B). However, HRAS expression caused an increase in PPARβ/δ reporter activity for both mouse and human isoforms, comparable to that observed with the positive control GW0742 (Figure 2C).

### 3.3. Bioinformatic Analyses Reveals Role of PPARβ/δ in Lipid Metabolism

**Figure 3:**
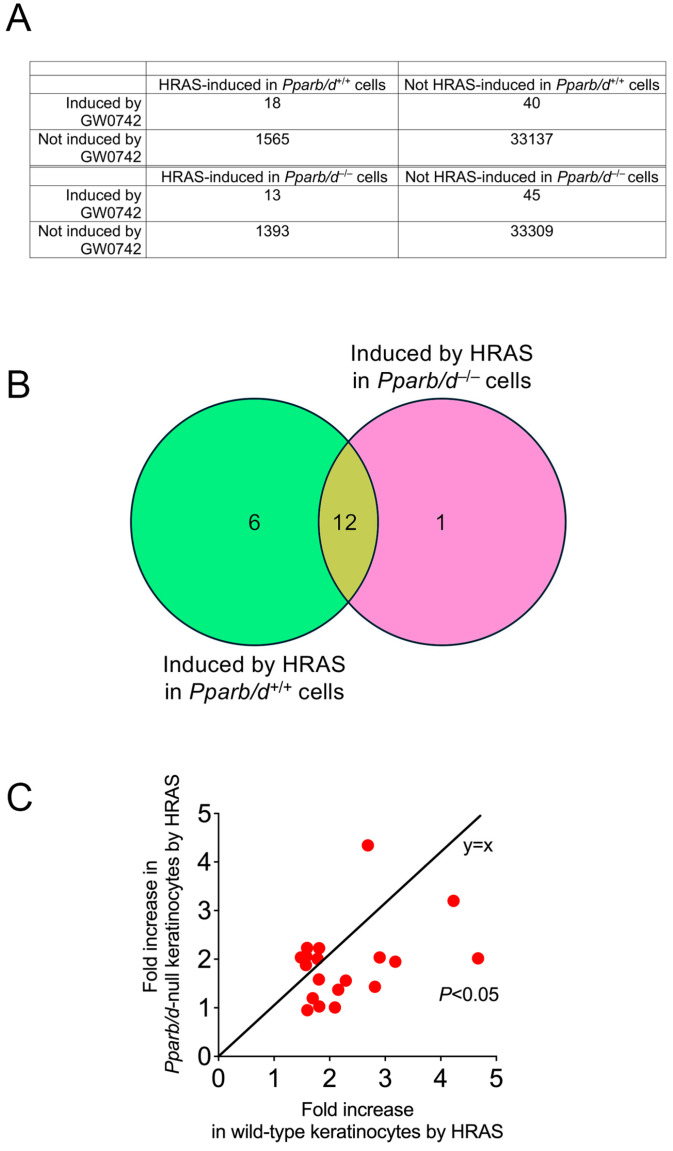
HRAS activation and GW0742 treatment induces common gene expression. (**A**) Overlap between the mRNAs that are induced by HRAS, and mRNAs are induced by the PPARβ/δ ligand GW0742 in primary keratinocytes. Only genes that are induced by ≥1.5 folds by either HRAS or GW0742 are included. *n* = 3 independent biological replicates. (**B**) A Venn diagram showing that 6 of 19 genes induced by GW0742 in wild-type keratinocytes are also induced by HRAS only in wild-type keratinocytes. In contrast, there was overlap in 12 genes between genotypes and treatments. (**C**) A plot of the fold change in genes in B induced by HRAS either in *Pparb/d* wild-type (*x*-axis) or *Pparb/d*-null keratinocytes (*y*-axis). Note that these genes are induced to a much higher fold by HRAS in wild-type keratinocytes compared to *Pparb/d*-null keratinocytes.

**Figure 4:**
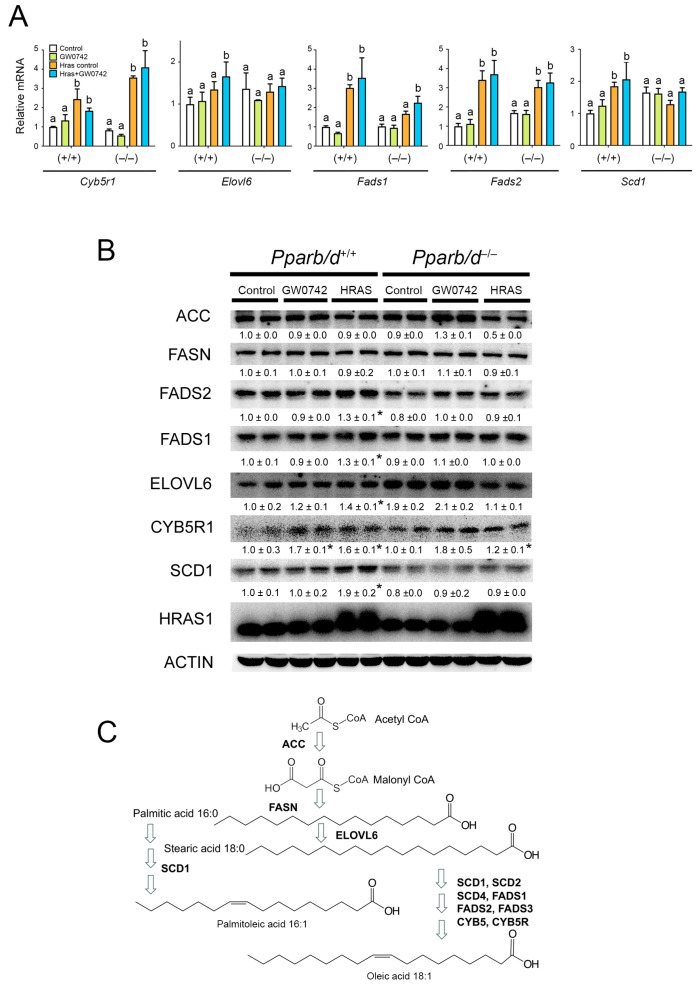
Activated HRAS increases expression of fatty acid synthesis genes. (**A**) qPCR of key fatty acid synthesis mRNAs in *Pparb/d* wild-type and *Pparb/d*-null keratinocytes treated with GW0742 or HRAS infection. *n* = 3–5 independent biological replicates. (**B**) Representative quantitative Western blot analysis of key fatty acid synthesis genes in *Pparb/d* wild-type and *Pparb/d*-null keratinocytes treated with GW0742 or HRAS infection. *n* = 4 independent biological replicates. (**C**) Diagram showing de novo fatty acid synthesis pathway influenced in these studies. Values represent mean ± SD. Values with different letters are significantly different, *p* ≤ 0.05. * Significantly different than control, *p* ≤ 0.05.

Bioinformatic analyses were performed using previously published microarray data from *Pparb/d* wild-type or *Pparb/d*-null keratinocytes and HRAS-expressing *Pparb/d* wild-type or *Pparb/d*-null keratinocytes, with or without the ligand activation of PPARβ/δ with GW0742 (GEO accession number GSE32498 [6,16]). Of particular interest were the mRNAs that were co-modulated by the PPARβ/δ ligand GW0742 and HRAS in both wild-type and *Pparb/d*-null keratinocytes (Figure 3A). There was overlap in the expression of twelve genes (*Acsl3*, *Lss*, *Tmtc2*, *Bnip3*, *Syngr1*, *Angptl4*, *Fosl1*, *Gm5246*, *Insig1*, *Fgfbp1*, *Phgdh*, and *Acsbg1*) that occurred (Figure 3B), but the expression of these genes was generally higher in HRAS-expressing wild-type keratinocytes as compared to HRAS-expressing *Pparb/d*-null keratinocytes (Figure 3C). Six genes were regulated specifically by PPARβ/δ (Figure 3B, *Scd1*, *Gdpd1*, *Erc2*, *Vwa8*, *AU18091*, and *Sna1*). Since SCD1 is rate-limiting for monounsaturated fatty acid synthesis, qPCR analyses of control or HRAS-expressing wild-type or *Pparb/d*-null keratinocytes were performed to examine other enzymes that regulate lipogenesis (Figure 4A). The expression of *Fads1*, *Fads2*, *Fads3*, *Cyb5r1*, and *Scd1* mRNAs was increased by HRAS in wild-type keratinocytes compared to controls, and this effect was mirrored by the ligand activation of PPARβ/δ with GW0742 in HRAS-expressing wild-type keratinocytes (Figure 4A). This effect was mitigated in *Pparb/d*-null HRAS-expressing keratinocytes and in response to GW0742 (Figure 3C and Figure 4A). Based on qPCR analyses, the expression of *Acc*, *Cyb5r2*, *Elovl6*, *Fasn*, and *Scd2* mRNAs was not differentially expressed in response to HRAS or GW0742 compared to controls. To determine whether the differential mRNA expression detected by qPCR analyses was similar for proteins, Western blot analyses were performed (Figure 4B). The ligand activation of PPARβ/δ with GW0742 did not influence the expression of any protein except CYB5R1 in wild-type keratinocytes, which was relatively higher, compared to the control (Figure 4B). Consistent with mRNA analyses, the relative expression of FADS1, FADS2, ELOLV6, CYB5R1, and SCD1 proteins was higher in HRAS-expressing wild-type keratinocytes, and this effect was not noted in HRAS-expressing *Pparb/d*-null keratinocytes (Figure 4B). These data suggest that the HRAS-dependent modulation of PPARβ/δ signaling could influence monounsaturated fatty acid synthesis and that these fatty acids may act as ligands for this receptor (Figure 4C).

### 3.4. HRAS Increases Oleic and Palmitoleic Acid Levels in Primary Keratinocytes and Activates PPARβ/δ

**Figure 5:**
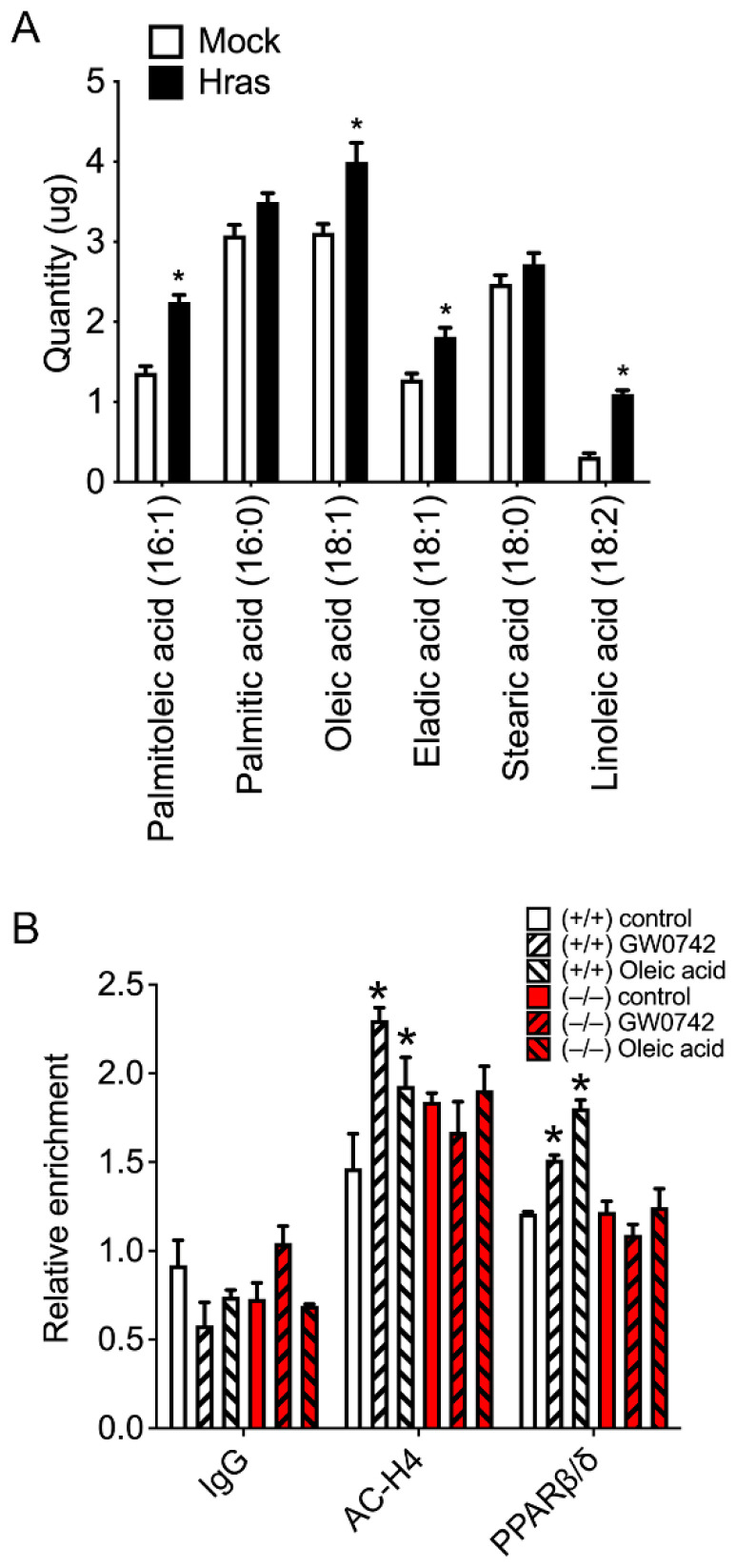
HRAS-expressing keratinocytes have higher palmitoleic and oleic acid levels, which are PPARβ/δ ligands. (**A**) Quantification of levels of key fatty acid in mock-infected or HRAS-infected *Pparb/d* wild-type keratinocytes by GC-MS. Data are compiled from three independent experiments. *n* = 5–6 independent biological replicates. (**B**) ChIP-qPCR analysis of Ac-H4 and PPARβ/δ binding to mouse *Angptl4* gene promoter in response to GW0742 or oleic acid treatment in *Pparb/d* wild-type or *Pparb/d*-null keratinocytes. *n* = 3 independent biological replicates. Values represent mean ± SD. * Significantly different than control, *p* ≤ 0.05.

**Figure 6:**
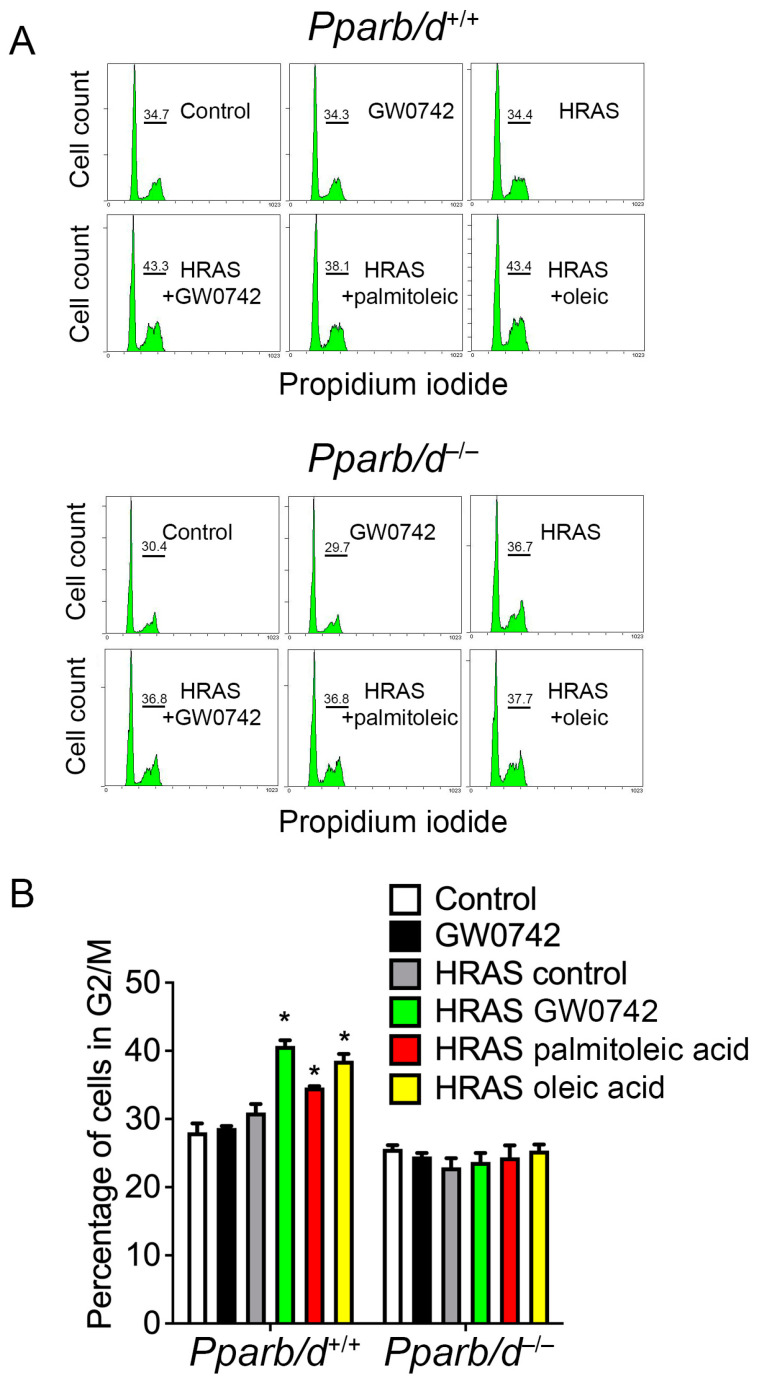
Palmitoleic and oleic acids induce G2/M arrest in HRAS-expressing keratinocytes through PPARβ/δ-dependent mechanism. Representative DNA histograms (**A**) and quantification of percentage of cells in G2/M phase, *n* = 3 independent biological replicates, (**B**) of mock-infected or HRAS-expressing *Pparb/d* wild-type or *Pparb/d*-null keratinocytes treated with GW0742, palmitoleic acid or oleic acid or 72 h. Representative peak of cells in G2/M phase shown above bar in each panel in (**A**). *n* = 3 independent biological replicates. Values in (**B**) represent mean ± SD. * Significantly different than control, *p* ≤ 0.05.

To determine whether changes in the expression of lipogenic proteins found in HRAS-expressing keratinocytes influenced the synthesis of lipids, GC-MS analysis was performed. The expression of HRAS in keratinocytes caused an increase in the amount of palmitoleic acid, oleic acid, eladic acid, and linoleic acid compared to controls (Figure 5A). ChIP analyses showed that oleic acid increased the occupancy of PPARβ/δ on the *Angplt4* PPRE in wild-type keratinocytes similar to that observed with the synthetic PPARβ/δ ligand GW0742, but this did not occur in *Pparb/d*-null keratinocytes (Figure 5B). As SCD1 is the rate-limiting enzyme catalyzing the synthesis of monounsaturated fatty acids (in particular for oleic and palmitoleic acids [37]), these observations collectively suggested that HRAS activation causes the synthesis of fatty acids that activate PPARβ/δ in keratinocytes. Since the ligand activation of PPARβ/δ results in the negative selection of cells expressing higher levels of the HRAS oncogene by inducing a mitotic block [16], cell cycle progression was examined. Consistent with previous studies, the ligand activation of PPARβ/δ in HRAS-expressing keratinocytes caused mitotic arrest at the G2/M phase of the cell cycle, and this effect was also observed in response to oleic acid or palmitoleic acid compared to controls (Figure 6). Importantly, this effect was not found in similarly treated HRAS-expressing *Pparb/d*-null keratinocytes (Figure 6).

### 3.5. Oleic Acid and Palmitoleic Acid Inhibit Hyperplasia in Keratinocytes

**Figure 7:**
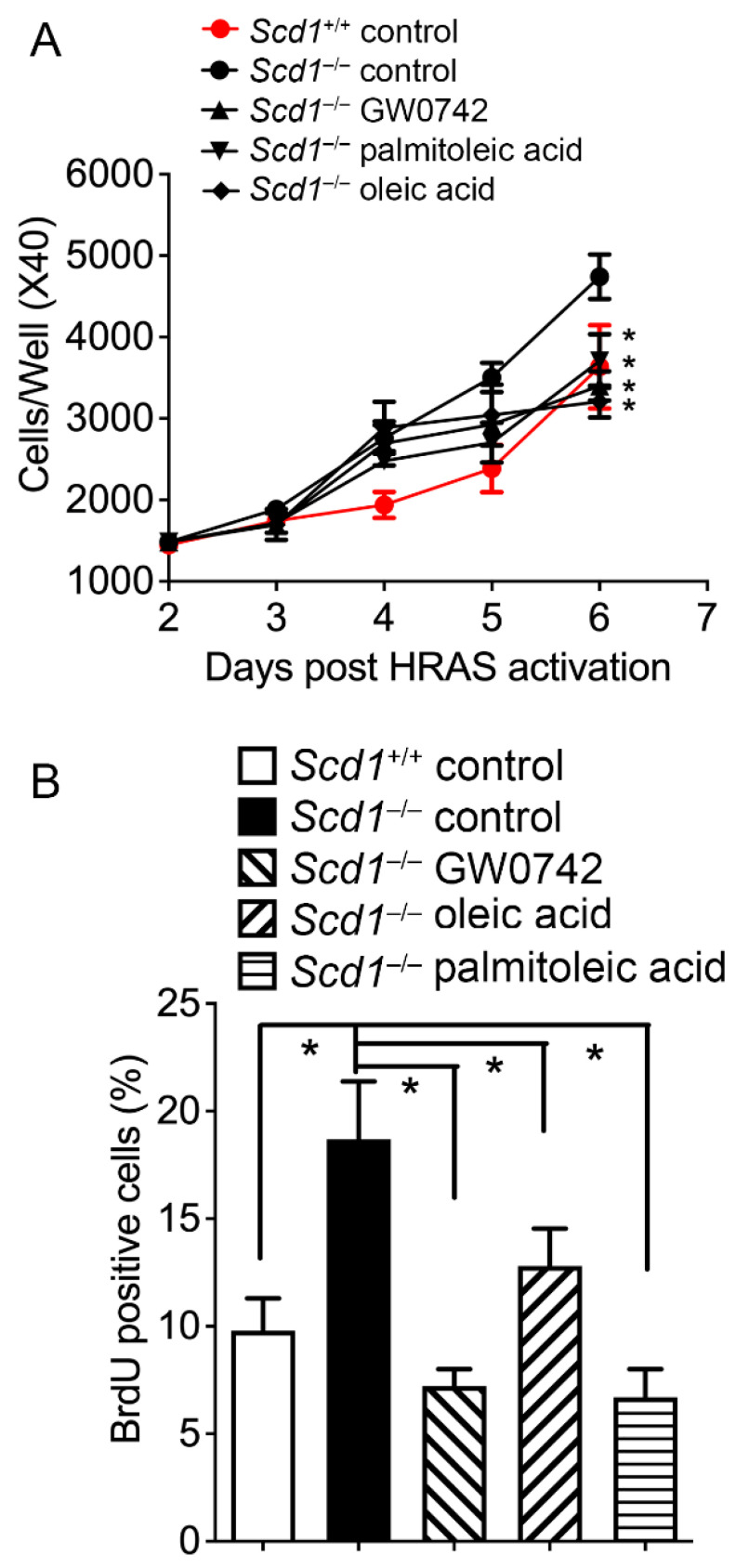
Palmitoleic and oleic acids rescue enhanced cell proliferation of HRAS-expressing keratinocytes resulting from *Scd1* ablation. (**A**) Cell counts of HRAS-expressing keratinocytes isolated from wild-type (*Scd1*^+/+^), *Scd1* knock-out (*Scd1*^−/−^), and *Scd1*^−/−^ treated with GW0742, palmitoleic acid, or oleic acid for 6 days post-HRAS expression. *n* = 3–6 independent biological replicates. (**B**) BrdU-positive cells were identified by immunofluorescence after 6 days of HRAS expression. *n* = 4–6 independent biological replicates. Values represent mean ± SD. * Significantly different than control, *p* ≤ 0.05.

**Figure 8:**
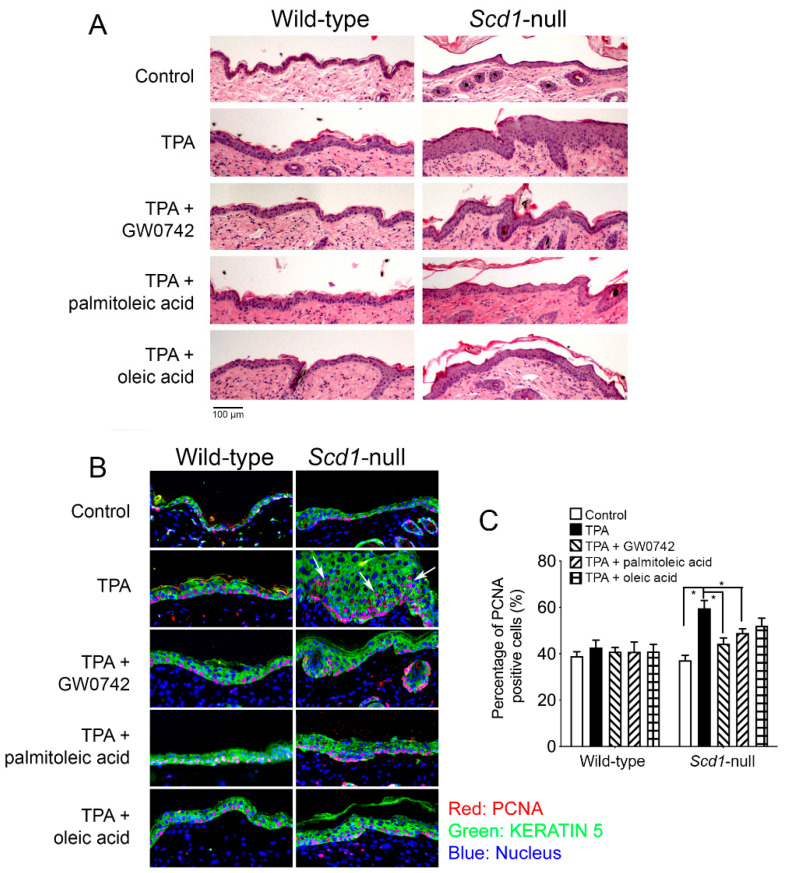
Palmitoleic and oleic acids rescue enhanced cell proliferation and hyperplasia resulting from *Scd1* ablation. (**A**) H&E staining of mouse skin from wild-type and *Scd1*^–/–^ mice treated topically once with TPA and with or without GW0742, palmitoleic acid, or oleic acid for 48 h. *n* = 3–10 independent biological replicates. The 100 µm scale in (**A**) is the same for images in (**B**). (**B**) Representative immunofluorescence of PCNA (red), keratin 5 (green), and DAPI (blue), and (**C**) quantification of percentage of PCNA-positive cells in skin of wild-type and *Scd1*^−/−^ mice treated with TPA and with or without GW0742, palmitoleic acid, or oleic acid for 48 h. Higher percentage of PCNA-positive cells shown with white arrows. *n* = 3–10 independent biological replicates. Values represent mean ± SD. * Significantly different than control, *p* ≤ 0.05.

HRAS-expressing *Scd1*-null keratinocytes exhibited enhanced cell proliferation compared to the wild-type control (Figure 7), a phenotype remarkably similar to *Pparb/d*-null keratinocytes [16]. Interestingly, GW0742, oleic acid and palmitoleic acid all rescued this phenotype in *Scd1*-null keratinocytes compared to controls (Figure 7). Similarly, topical treatment with TPA caused epidermal hyperplasia in wild-type mice, and this effect was exacerbated in *Scd1*-null mice compared to the control (Figure 8). Similar to that observed with in vitro primary keratinocytes, this phenotype of the TPA-treated *Scd1*-null mouse skin was remarkably similar (Figure 8) to that observed in TPA-treated *Pparb/d*-null mouse skin that exhibited enhanced epidermal hyperplasia compared to controls [19]. More importantly GW0742, palmitoleic acid, and oleic acid inhibited TPA-induced hyperplasia in *Scd1*-null mice (Figure 8). Collectively, these results suggest that the supplementation of exogenous monounsaturated fatty acids can rescue the hyperplasia phenotypes of *Scd1*-null mice, likely via the ligand activation of PPARβ/δ.

### 3.6. PPARβ/δ-Dependent Inhibition of UVB-Induced Skin Cancer with Oleic Acid and Synthetic Ligand

**Figure 9:**
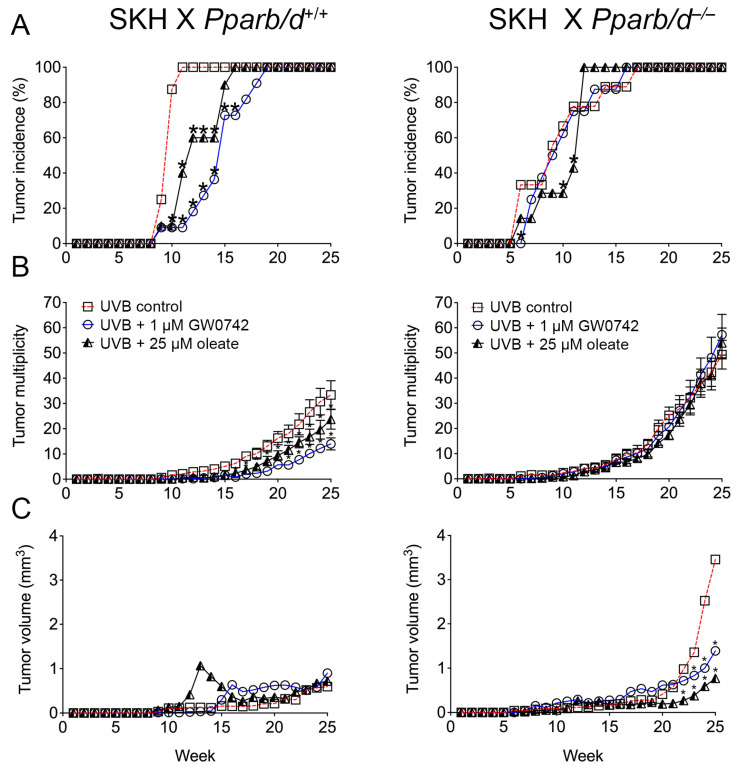
Ligand activation of PPARβ/δ with oleic acid or GW0742 inhibits UVB-induced skin cancer. SKH mice were crossed with *Pparb/d* wild-type or *Pparb/d*-null mice and irradiated with or without topical application of PPARβ/δ ligands after irradiation. Onset of tumor formation (**A**), number of tumors per mouse (**B**), and average tumor size (**C**) were measured weekly. *n* = 7–11 independent biological replicates. Each point represents mean ± S.D. * *p* < 0.05 compared with control.

The ligand activation of PPARβ/δ with GW0742 is chemopreventive against chemically induced skin cancer [13,14,15]. This effect requires PPARβ/δ as the inhibition of malignant conversion was only noted in ligand-treated wild-type mice but not in ligand-treated *Pparb/d*-null mice [13,14,15]. The chemopreventive effect of the ligand activation of PPARβ/δ on the tumor incidence, tumor multiplicity, and tumor volume of UVB-induced skin tumors was examined in SKH-1 mice crossed with *Pparb/d*-wild-type or *Pparb/d*-null mice. The onset of UVB-induced tumor formation was delayed in SKH1 X *Pparb/d^+/+^* mice by either the topical application of oleic acid or GW0742 as compared to controls, and this effect was not observed in GW0742-treated or SKH1 X *Pparb/d*^−/−^ mice (Figure 9A). The incidence of UVB-induced tumor formation was slightly delayed in SKH1 X *Pparb/d*^−/−^ mice by the topical application of oleic acid as compared to controls, but this effect was not observed in GW0742-treated or control SKH1 X *Pparb/d*^−/−^ mice (Figure 9A). The ligand activation of PPARβ/δ with either GW0742 or oleic acid inhibited tumor multiplicity in SKH1 X *Pparb/d^+/+^* mice from weeks 17 to 25, and this effect was not observed in similarly treated or SKH1 X *Pparb/d*^−/−^ mice (Figure 9B). Tumor multiplicity was higher during the last 5 weeks of treatment in all groups of SKH1 X *Pparb/d*^−/−^ mice compared to in SKH1 X *Pparb/d^+/+^* mice (Figure 9B). The average tumor volume was not influenced by the ligand activation of PPARβ/δ with either GW0742 or oleic acid in SKH1 X *Pparb/d^+/+^* mice (Figure 9C). The average tumor volume was greater from weeks 23 to 25 in SKH1 X *Pparb/d*^−/−^ mice compared to control SKH1 X *Pparb/d^+/+^* mice (Figure 9C). Interestingly, the average tumor volume was lower from weeks 23 to 25 in SKH1 X *Pparb/d*^−/−^ mice in response to treatment with either GW0742 or oleic acid compared to control SKH1 X *Pparb/d*^−/−^ mice (Figure 9C).

## 4. Discussion

The premise for these studies is based on seminal work by the Wahli research group in 2001 suggesting that keratinocytes are a source of PPARβ/δ ligands [18]. The results from the present studies extend this original observation by showing that oleic acid and palmitoleic acid are increased in keratinocytes by HRAS, and this modulates PPARβ/δ activities in these cells. This is consistent with the observation that TPA causes the release of fatty acids in keratinocytes [33]. That endogenous ligands exist and dynamically modulate gene expression in keratinocytes is strongly supported by previous studies showing that the genetic silencing of *Pparb/d* in keratinocytes causes alterations in the constitutive expression of hundreds of PPARβ/δ target genes [6]. Additionally, increasing the basal expression of PPARβ/δ is known to inhibit the size and growth of tumors derived from human skin, neuroblastoma, breast cancer, melanoma, and testicular cancer cell lines implanted in mice, in the absence of exogenous ligands (reviewed in [12,38]). The constitutive, nuclear localization of PPARβ/δ is also relatively high in epithelial cells and PPARβ/δ co-immunoprecipitates with its heterodimerization partner, RXR [9]. These observations strongly support the hypothesis that PPARβ/δ is constitutively active and has critical roles in normal, basal keratinocyte physiology including the induction of terminal differentiation [39,40,41]. This appears particularly true for epithelial cells such as those found in the intestine or skin where the basal expression of PPARβ/δ is relatively high compared to other tissues in mice and humans [9,10].

SCD1 is the rate-limiting enzyme that catalyzes the synthesis of monounsaturated fatty acids including oleic and palmitoleic acids [37]. The results from the present studies show that high HRAS activity in keratinocytes caused a greater expression and activity of SCD1, and this effect requires PPARβ/δ. This is consistent with the higher levels of palmitoleic acid and oleic acid found in keratinocytes by increasing HRAS activity. As the ligand activation of PPARβ/δ causes the negative selection of cells expressing higher levels of HRAS by inducing a mitotic block at the G2/M phase of the cell cycle [16], it is critical to note that oleic acid, palmitoleic acid, and GW0742 prevented an increase in the percentage of cells remaining in the G2/M phase of the cell cycle induced by TPA in *Scd1*-null keratinocytes. Similarly, the epidermal hyperplastic response induced by TPA was exacerbated in *Scd1*-null mice, a phenotype that is remarkably similar to the phenotype noted in TPA-treated *Pparb/d*-null mice [15,19,40,42]. Importantly, palmitoleic acid, oleic acid, and GW0742 all reversed the TPA-induced hyperplasia in *Scd1*-null epidermis. These observations collectively support the hypothesis that HRAS-expressing cells synthesize PPARβ/δ ligands to inhibit the cell proliferation of mutant cells, likely the result of inducing terminal differentiation. Future studies should consider an examination of SCD1 inhibitors, as well as the influence of other monounsaturated fatty acids, to complement and extend the present studies.

The observations made in *Scd1*-null mouse skin support previous studies demonstrating that the ligand activation of PPARβ/δ with a synthetic ligand inhibits chemically induced skin carcinogenesis [13,14,15]. Interestingly, a higher expression of SCD1 is negatively associated with cancer cell growth and progression [43], and altered levels of monounsaturated fatty acids produced by SCD1 can both inhibit or promote cancer cell survival [43]. In contrast, UV exposure causes the inhibition of SCD1 expression and lower levels of fatty acids in human skin [44]. This is consistent with the present results showing that oleic and palmitoleic acid can act to inhibit UV-induced non-melanoma skin cancer. Given the growing incidence of non-melanoma skin cancer [45], further studies should examine whether the dietary administration of these lipids may prevent this disease. Further studies are also needed to delineate how SCD1-dependent regulation impacts PPARβ/δ function (and vice versa) in other cancers. These observations are also consistent with several two-stage chemical carcinogenesis studies showing that the ligand activation of PPARβ/δ inhibits skin carcinogenesis caused by the topical administration of dimethylbenzanthracene (DMBA) and TPA [13,14,15]. As the malignant conversion of tumors in the DMBA/TPA model requires the mutation of *HRAS*, the present studies suggest that the production of PPARβ/δ ligands during the time course of a two-stage chemical carcinogenesis bioassay may contribute to the observed inhibition noted in the previous studies [13,14,15]. Whether this mechanism reflects a negative feedback loop to prevent the proliferation of mutant keratinocytes requires additional evaluation. Since UVB-induced skin cancer is dependent on mutations in the TP53 gene, the sensitivity of TP53 mutant cells to the effects of the ligand activation of PPARβ/δ should be further examined. Another study found that UV-induced skin carcinogenesis was mitigated in SKH1 X *Pparb/d*-null compared to SKH1 X *Pparb/d* wild-type mice and that this effect was modulated by the regulation of SRC expression [46]. The results from the present studies cannot be compared to the former studies for several reasons. First, the UV light source used for the latter studies used both UVB and UVA [46], whereas the present studies used only UVB and eliminated the impact of UVA/UVC by using a filter. This is important to note because UVB is thought to be the most predominant source of solar radiation that causes cyclobutane pyrimidine dimers and pyrimidine (6-4) photoproducts [47,48,49] and is more commonly used for skin carcinogenesis bioassays. Second, the *Pparb/d*-null mice used for both studies used different approaches to silence PPARβ/δ expression. It remains possible that an off-target event(s) in the two transgenic lines underlie this difference. Lastly, important symbiotic roles of bacteria, viruses, and fungi are being revealed that may impact many routes of exposure. Thus, it is also possible that differences in the skin microbiome impacts results from these two studies. Further work is needed to clarify these issues.

## Data Availability

The data presented in this study are available on request from the corresponding author. The data are not publicly available due to the author’s preference.

## Supplementary Materials

The following supporting information can be downloaded at: https://www.mdpi.com/article/10.3390/biom14060606/s1. Original Western Blots.
