# Supplementary figures and images for: Activation of Peroxisome Proliferator-Activated Receptor-β/δ (PPARβ/δ) in Keratinocytes by Endogenous Fatty Acids
Source: Biomolecules. 2024 May 21;14(6):606. doi: 10.3390/biom14060606 (PMC11201440; doi:10.3390/biom14060606)

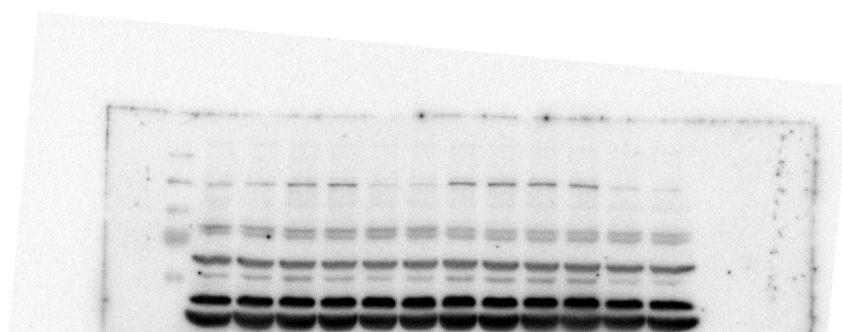

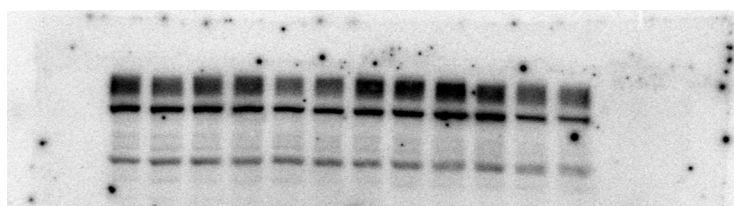

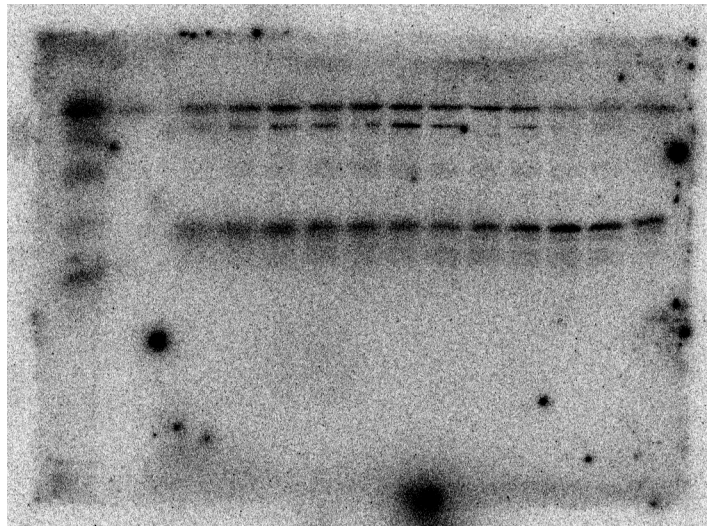

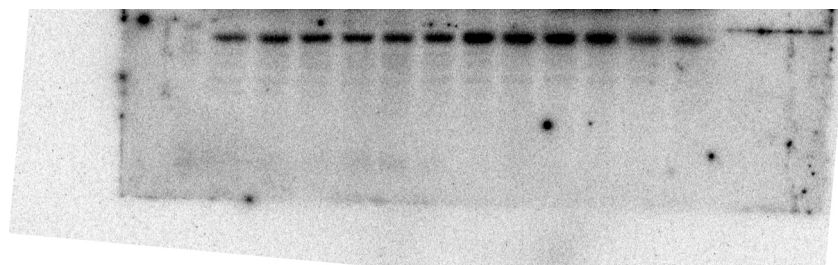

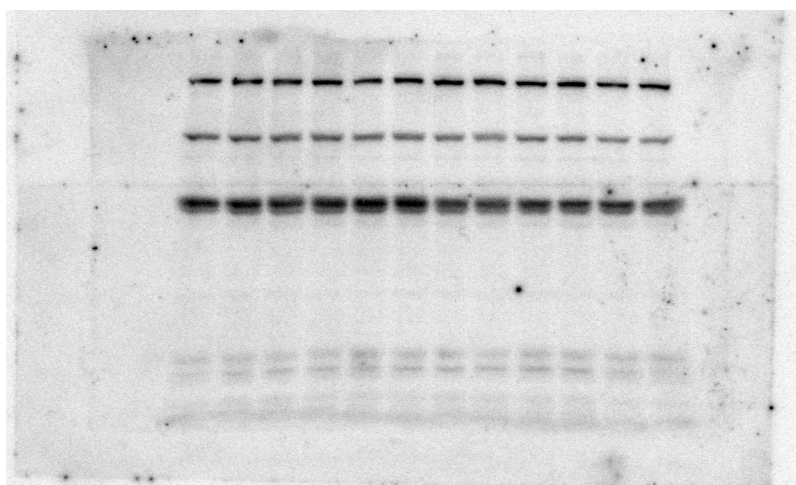

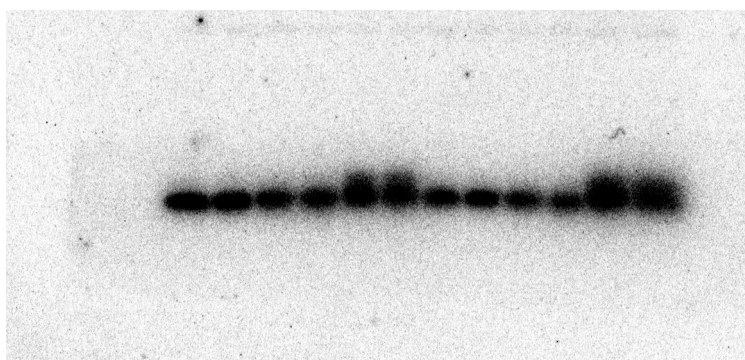

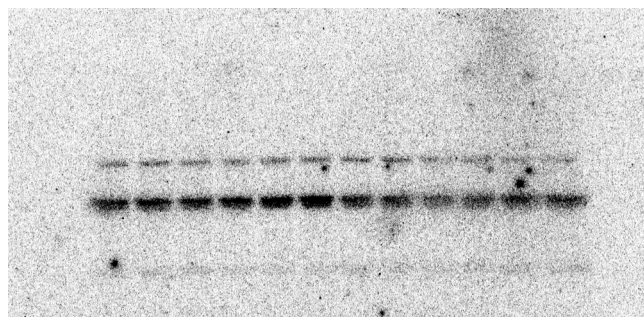

Supplement: Supplementary file 1 [file biomolecules-14-00606-s001.zip › biomolecules-2992249-supplementary.pdf]
